# Unraveling Bonding Mechanisms and Electronic Structure of Pyridine Oximes on Fe(110) Surface: Deeper Insights from DFT, Molecular Dynamics and SCC-DFT Tight Binding Simulations

**DOI:** 10.3390/molecules28083545

**Published:** 2023-04-18

**Authors:** Hassane Lgaz, Han-seung Lee, Savaş Kaya, Rachid Salghi, Sobhy M. Ibrahim, Maryam Chafiq, Lahcen Bazzi, Young Gun Ko

**Affiliations:** 1 Innovative Durable Building and Infrastructure Research Center, Center for Creative Convergence Education, Hanyang University ERICA, 55 Hanyangdaehak-ro, Sangrok-gu, Ansan-si 15588, Gyeonggi-do, Republic of Korea; hlgaz@hanyang.ac.kr; 2 Department of Architectural Engineering, Hanyang University-ERICA, 55 Hanyangdaehak-ro, San-grok-gu, Ansan-si 15588, Gyeonggi-do, Republic of Korea; 3 Department of Pharmacy, Health Services Vocational School, Sivas Cumhuriyet University, Sivas 58140, Turkey; savaskaya@cumhuriyet.edu.tr; 4 Laboratory of Applied Chemistry and Environment, Applied Science National School (ENSA), University Ibn Zohr, P.O. Box 1136, Agadir 80000, Morocco; r.salghi@uiz.ac.ma; 5 Laboratoire de Génie Industriel, de l’Énergétique et de l’Environnement (LGI2E), SupMTI, Rabat 10000, Morocco; bazzilahcen@gmail.com; 6 Department of Biochemistry, College of Science, King Saud University, P.O. Box 2455, Riyadh 11451, Saudi Arabia; sobhy.yakout@gmail.com; 7 Materials Electrochemistry Group, School of Materials Science and Engineering, Yeungnam University, Gyeongsan 38541, Gyeongsangbuk-do, Republic of Korea

**Keywords:** pyridine, oxime, DFT, SCC-DFTB, computational methods, corrosion inhibitor, adsorption, density of states

## Abstract

The development of corrosion inhibitors with outstanding performance is a never-ending and complex process engaged in by researchers, engineers and practitioners. The computational assessment of organic corrosion inhibitors’ performance is a crucial step towards the design of new task-specific materials. Herein, the electronic features, adsorption characteristics and bonding mechanisms of two pyridine oximes, namely 2-pyridylaldoxime (2POH) and 3-pyridylaldoxime (3POH), with the iron surface were investigated using molecular dynamics (MD), and self-consistent-charge density-functional tight-binding (SCC-DFTB) simulations. SCC-DFTB simulations revealed that the 3POH molecule can form covalent bonds with iron atoms in its neutral and protonated states, while the 2POH molecule can only bond with iron through its protonated form, resulting in interaction energies of −2.534, −2.007, −1.897, and −0.007 eV for 3POH, 3POH^+^, 2POH^+^, and 2POH, respectively. Projected density of states (PDOSs) analysis of pyridines–Fe(110) interactions indicated that pyridine molecules were chemically adsorbed on the iron surface. Quantum chemical calculations (QCCs) revealed that the energy gap and Hard and Soft Acids and Bases (HSAB) principles were efficient in predicting the bonding trend of the molecules investigated with an iron surface. 3POH had the lowest energy gap of 1.706 eV, followed by 3POH^+^ (2.806 eV), 2POH^+^ (3.121 eV), and 2POH (3.431 eV). In the presence of a simulated solution, MD simulation showed that the neutral and protonated forms of molecules exhibited a parallel adsorption mode on an iron surface. The excellent adsorption properties and corrosion inhibition performance of 3POH may be attributed to its low stability compared to 2POH molecules.

## 1. Introduction

Pyridines are one of the most prevalent heterocyclic structural scaffolds and have a broad spectrum of applications in different fields such as pharmaceuticals, organic synthesis, dyes, additives, agrochemicals, and materials science, among others [1,2]. This unique aromatic ring forms the core of many pesticides, drugs, and natural products such as alkaloids, coenzymes, and vitamins [3,4]. Driven by the demands of these various applications, numerous synthesis approaches have been developed along with traditional amine and carbonyl condensation processes [5,6]. Another interesting field where these heterocyclic compounds have a wide range of applications is the corrosion mitigation of metals in different corrosive mediums [7,8,9,10,11].

Pyridine derivatives are a viable option for inhibiting corrosion of iron alloys, copper, and aluminum [12]. The importance of these heterocycles in corrosion inhibition studies comes from their electron-rich structures and the presence of several active sites. It is well-documented that organic corrosion inhibitors act by adsorption on metal surfaces, forming a protective barrier that prevents further corrosion [13]. The molecular structure of the corrosion inhibitor is one of the factors that has a great influence on the effectiveness of the adsorption process. Organic compounds containing heteroatoms such as O, N, and S, and/or functional groups such as methyl, methoxy, dimethylamine, and aromatic rings, etc., act as excellent corrosion inhibitors [12,14,15,16]. These heteroatoms and functional groups constitute the main bonding sites with metallic materials through transfer of their non-bonding and π-electrons into the vacant *d*-orbitals of the metallic surface.

Numerous experimental studies have been conducted to assess the corrosion inhibition performance of pyridine derivatives. Despite their enduring importance in providing essential information about inhibition efficiency and general corrosion protection mechanisms, experimental studies are time and resource intensive, and are unable to reveal insights into the direct interactions between molecules and metal surfaces. In an attempt to overcome this limitation, researchers have proposed several computational approaches such as quantum chemical calculations and molecular dynamics simulations to assess the reactivity of individual compounds and their interactions with metallic surfaces [17,18,19]. In this regards, Ansari et al. [20] have reported experimental and quantum chemical studies on two pyridine derivatives for N80 steel in 15 wt.% HCl. The two compounds, namely 2-amino-6-(2,4-dihydroxyphenyl)-4-(4-methoxyphenyl)nicotinonitrile (ADP) and 2-amino-4-(4-methoxyphenyl)-6-phenylnicotinonitrile (AMP) had corrosion inhibition efficiencies of 90% and 86% at 200 mg/L, respectively. In addition to electrochemical and surface characterization techniques, the authors reported quantum chemical calculations for neutral and protonated molecules. They concluded that ADP had a higher highest occupied molecular orbital energy compared to AMP because of its electron donor hydroxyl group on the phenyl ring. The authors also reported that the ADP had a lower energy gap value, suggesting its strong ability to be adsorbed into the metal surface. Quantum chemical studies of protonated molecules revealed that protonated molecules had a higher electron donating tendency compared to neutral ones. The same research team have conducted another study on three pyridines, namely 2-amino-6-methoxy-4-(4-methoxylphenyl) pyridine-3,5-dicarbonitrile (PC-1), 2-amino-6-methoxy-4-(4-methylphenyl) pyridine-3,5-dicarbonitrile (PC-2), and 2-amino-6-methoxy-4-phenylpyridine-3,5-dicarbonitrile (PC-3) for mild steel in 1.0 mol/L HCl [21]. The authors attributed the fact that PC-1 had the highest inhibition performance to it having the lowest energy gap and the highest global softness. More recently, Berisha [22] studied the corrosion inhibition properties of pyridine, 2-amino-5-chloropyridine, 2-amino-3,5-dichloropyridine, and 2-amino-3-benzyloxypyridine using experimental density-functional theory and molecular dynamics simulation for mild steel in perchloric acid solution. The author reported that the inhibition efficiency of the investigated pyridines increased from 27% for pyridine, to 68% for the 2-amino-5-chloropyridine at 0.05 mol/L. From theoretical studies, the author concluded that the tested pyridines had widespread reactive sites that promoted pyridines’ adsorption into the mild steel surface, which adsorption was found to be in a flat-lying manner on the Fe(110) surface as revealed by molecular dynamics simulation. These investigations and others [14,15,16] provided useful insights into the ability of pyridine derivatives to inhibit metal corrosion in different mediums. However, the theoretical approach based on the reactivity of individual molecules has been found to be limited in predicting the corrosion inhibition performance and adsorption characteristics of compounds on metals [23,24]. Molecular dynamics, on the other hand, are one of the recent computational methods most widely used to simulate inhibitor-metal interactions [25]. One of the main advantages of molecular dynamics simulation is its ability to model inhibitor adsorption on metal surfaces in the presence of a simulated corrosive solution, which can provide interesting knowledge about the inhibitor’s adsorption configuration and its adsorption competitiveness. Nevertheless, despite the advanced progress made to develop more reliable force fields, this approximated molecular mechanics (MM) method is limited by some drawbacks such as an inability to simulate fundamentally quantum processes such as bond-formation and bond-breaking upon adsorption of molecules on metal surfaces [26,27].

Ab initio computational methods are emerging as an advanced set of tools for the analysis of the adsorption characteristics of corrosion inhibitors on metal surfaces [28,29]. For instance, ab initio DFT simulation of inhibitor–metal interactions can reveal deeper insights into stable adsorption geometries, bonding mechanisms, and the adsorption strength and electronic properties of interacting species, which are inaccessible from the above-mentioned computational methods [28,29]. Given the large size of many chemical systems, continuous efforts have been made to develop approximated methods to reduce computational complexity. The self-consistent-charge density-functional tight-binding (SCC-DFTB) is a density-functional theory (DFT)-based approximated method [27]. It is an approximation that assumes a second-order expansion of the Kohn–Sham Density Functional Theory and its accuracy in predicting structural and electronic properties is similar to ab initio calculations, but it is ∼100–1000 times faster and also suitable for large systems [27,30,31]. Therefore, implementation of this computational method along with molecular dynamics simulation would be ideal to comprehensively investigate the reactivity and adsorption characteristics of corrosion inhibitors from different theoretical perspectives.

Two pyridine oxime derivatives, namely 2-pyridylaldoxime (2POH) and 3-pyridylaldoxime (3POH), have been previously reported as corrosion inhibitors for carbon steel in 1.0 mol/L HCl solutions [32]. Authors have found that tested oximes exhibited excellent corrosion inhibition properties with inhibition efficiencies of 79% and 94% for 2POH and 3POH, respectively. Electrochemical measurements revealed that the addition of both compounds to HCl solution led to a significant decrease in double-layer capacitance as well as to a reduction in both anodic and cathodic corrosion reactions, suggesting that the tested pyridine oximes were effectively adsorbed on the carbon steel surface.

Motivated by the lack of a comprehensive investigation covering electronic and reactivity features and the similar lack of investigation on the underlying bonding mechanisms of the investigated pyridine oximes on carbon steel surfaces, the present study reports the computational assessment of 2-pyridylaldoxime (2POH) and 3-pyridylaldoxime (3POH) through molecular dynamics (MD) and self-consistent-charge density-functional tight-binding (SCC-DFTB) simulations. QCCs were carried out to investigate the structural and electronic properties of the investigated pyridine oximes, however they are reported as complementary studies. MD simulation was performed to acquire insights into the adsorption configuration of molecules on an iron surface in the absence and presence of corrosive particles. Furthermore, SCC-DFTB simulations of inhibitor–iron interactions were conducted to evaluate the most stable adsorption geometries and interaction energies. The underlying bonding mechanisms were assessed through projected density of states analysis. All investigations were carried out on neutral and protonated pyridine oximes.

## 2. Results and Discussion

### 2.1. SCC-DFTB Simulations

#### 2.1.1. Geometries and Energies

**Figure 1:**
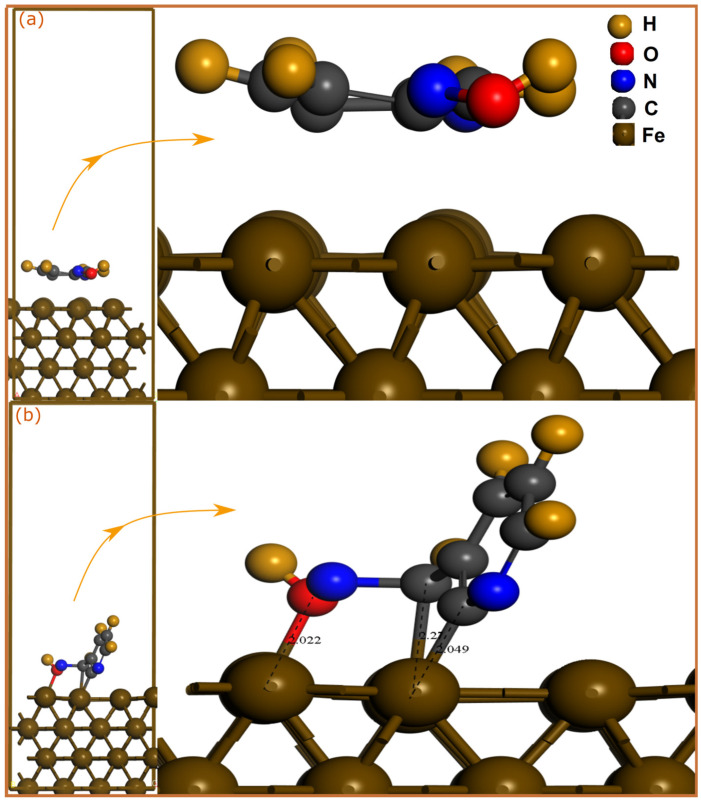
SCC-DFTB-optimized adsorption geometries of neutral and protonated 2POH molecules on Fe(110) surface; (**a**) neutral and (**b**) protonated forms. Bond distances are in Å.

**Figure 2:**
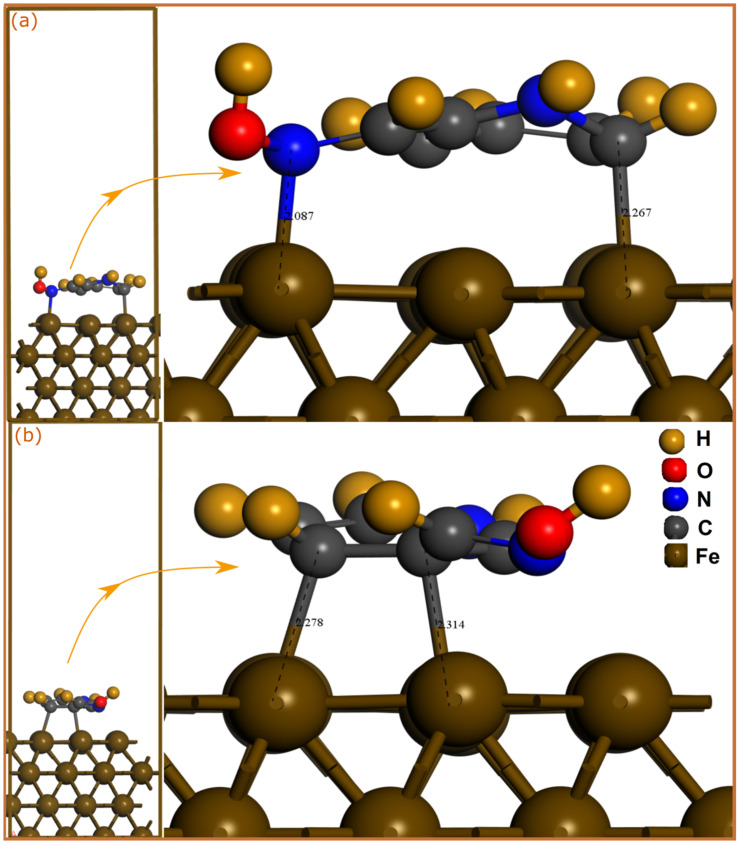
SCC-DFTB-optimized adsorption geometries of neutral and protonated 3POH molecules on Fe(110) surface; (**a**) neutral and (**b**) protonated forms. Bond distances are in Å.

SCC-DFTB simulations are a highly powerful and accurate theoretical tool for the atomic characterization of interfaces. The utilization of this semi-empirical method is also assured by the fact that, being a suitable for a particular task, it results in physically meaningful results with excellent accuracy [27]. In order to analysis the adsorption mechanism of pyridine oximes on a carbon steel surface, the adsorption geometries of neutral and protonated molecules on Fe(110) surface were optimized by SCC-DFTB simulation as shown in Figure 1 and Figure 2.

Inhibitor molecules can be adsorbed on the iron surface through physical and/or chemical interactions. An interatomic distance within the sum of the covalent radii of interacting atoms mainly indicates a chemical bonding, while physical interactions usually occur at interatomic distances beyond 3 Å. Looking at optimized adsorption geometries of neutral molecules, one can notice that 2POH and 3POH have a very different adsorption mode on a Fe(110) surface. The 2POH molecule (Figure 1a) approaches the iron surface but with a long adsorption distance, therefore no chemical bonding is formed. The molecule is stabilized in a parallel orientation to the surface, which might lead to the formation of physical interactions between the N atoms and iron surface. However, in contrast to 2POH, it can be observed that the 3POH molecule (Figure 2a) is stabilized in a nearly vertical adsorption mode on the Fe(110) surface, with the formation of three bonds with iron atoms. C(4) and C(7) form bonds with Fe atoms with distances of 2.049 Å and 2.27 Å, respectively, while the O(13)-Fe distance is 2.022 Å. For protonated molecules, the bonding situation is totally different for the 2POH^+^ molecule (Figure 1b). In this case, the 2POH^+^ molecule is parallelly stabilized on the Fe(110) surface through formation of two bonds, Fe-C(6) and Fe-N(8) with length distances of 2.267 Å and 2.087 Å, respectively. In the case of the 3POH^+^ molecule (Figure 2b), it is the adsorption mode that is different compared with the neutral one, with the formation of only two Fe-C(2) and Fe-C(3) bonds in a planar disposition on the metal surface. It can be inferred from these results that the 3POH molecule has a higher affinity to iron atoms in its neutral and protonated forms, while the 2POH tends to bond with iron atoms only through its protonated form.

To aquire basic information about the nature of the bonds formed between pyridine oximes and iron atoms, the sum of the covalent radii of interacting atoms can be compared with the bond distance [33]. It has been reported that the sum of the covalent radii for Fe-C (r_C_ + r_Fe_), Fe-N (r_N_ + r_Fe_) and Fe-O (r_O_ + r_Fe_) are 2.08, 2.07 and 1.98 Å, respectively [34]. By comparison with the calculated bond distances, one can notice that Fe-C, Fe-O and Fe-N bond lengths are all within the sum of the corresponding covalent radii, suggesting that pyridine oxime molecules might have strong chemical interactions with the iron surface.

The adsorption strength of pyridine oximes on the iron surface can be characterized by the interaction energies analyzed from the optimized adsorption geometries. As shown in Table 1, the neutral form of the 3POH molecule exhibits the highest magnitude of interaction energy, reinforcing the fact that it makes the strongest contribution to the adsorption of the 3POH compound and it is followed by its protonated form. Both neutral and protonated forms of 3POH have a strong negative interaction energy of −2.534 and −2.007 eV, respectively, attributable to their thermodynamically favorable adsorption properties and affinity to iron atoms [33,35]. In the case of the 2POH compound, although the neutral molecule has a favorable adsorption ability on a Fe(110) surface as evidenced by its negative interaction energy, it is just −0.007 eV because of the long adsorption distance and absence of chemical bonds. However, the protonated form seems to be more thermodynamically stable than its neutral counterpart, with an interaction energy of −1.897 eV. Together, these results suggest that the 3POH compound would have strong adsorption characteristics and affinity to iron atoms compared with 2POH. This conforms to the previously reported experimental results, which showed that 3POH had the highest corrosion inhibition performance; it had an inhibition efficiency of 94% that outperformed the 2POH compound by 15%. Given the reported results, it can be concluded that the molecules investigated are principally adsorbing on the iron surface through chemical and van der Waals dispersion interactions. Long-range weak dispersion interactions play a very important role in the interaction of molecules with different surfaces [36]. These interactions can compete with chemical interactions, which involve the sharing or transfer of electrons between functional groups on the pyridine molecules and the iron surface. However, at an experimental level, interactions between pyridine molecules and iron surface are certainly more complicated, and other interactions such as coulomb interactions would also contribute to the adsorption strength of inhibitor molecules on the surface, particularly protonated molecules in the presence of intermediate species such as pre-adsorbed chlorides [37,38,39]. Additionally, aldoxime is known for its electron-withdrawing effect; it can withdraw electrons from the pyridine ring, thus decreasing its electron density. This effect would be more pronounced when the aldoxime is at position 2, because its electron-withdrawing effect will be higher, therefore limiting the ability of the pyridine ring to coordinate with iron atoms. It can, therefore, be suggested that substitution of pyridine ring by electron withdrawing groups should not be carried out at position 2. An analysis of optimized adsorption geometries via projected density of states would reveal useful information about the bonding mechanisms.

#### 2.1.2. Projected Density of States

**Figure 3:**
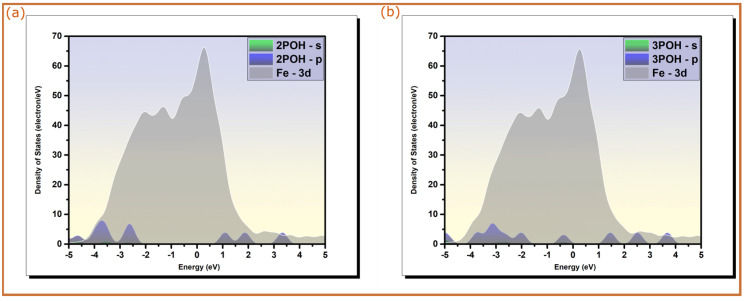
Projected density of states of isolated pyridine molecules on Fe(110) surface (7 Å above the top iron layer); (**a**) 2POH and (**b**) 3POH. Fermi energy (EF) is chosen as the zero energy level.

**Figure 4:**
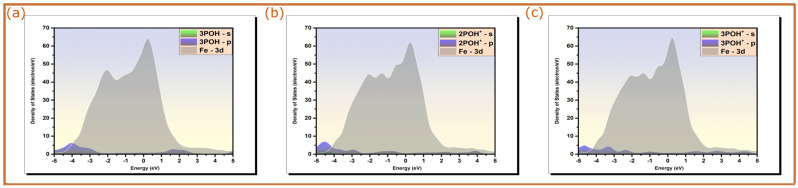
Projected density of states of pyridine molecules adsorbed on Fe(110) surface; (**a**) 3POH, (**b**) 2POH^+^, and (**c**) 3POH^+^. Fermi energy (EF) is chosen as the zero energy level.

The electronic structure of the optimized adsorption geometries of pyridine oximes on a Fe(110) surface was investigated by plotting the density of states projected on s and p orbitals of the molecules and 3d orbitals of the metallic fragment for both neutral and protonated forms of 3POH and the protonated form of 2POH. The changes in spectral shapes of PDOS prior and after the adsorption of pyridine oximes on a Fe(110) surface can be indicative of their bonding with iron atoms. Figure 3 illustrates the PDOS plots of molecules in their isolated states (7 Å above the top iron layer), while Figure 4 shows the PDOSs of neutral and protonated forms of 2POH and 3POH adsorbed on the iron surface along, with those of iron orbitals.

From the results depicted in Figure 3, it can be seen that before the adsorption process, sharp and intense peaks are observed in PDOS plots at various energy levels. A large part of these peaks lies within the −5/5 eV energy range, where the 3d bands of iron are localized [40]. This would favor the direct overlap between s and p orbitals of molecules with vacant *d*-orbitals of iron atoms. It should be noted that the obtained energy range of iron is consistent with several previous studies on the adsorption of organic molecules on iron surface [41,42]. The PDOS plots of the optimized adsorption structures (Figure 4) have broadened molecular peaks and most of those peaks shown before adsorption disappear. Additionally, one can notice a slight shift in energy to lower values after adsorption. This behavior is likely ascribed to hybridization interactions [40]. The inhibitors’ molecular orbitals will likely hybridize with metal *d*-states forming hybrid orbitals through the charge transfer process, which leads to significant modifications of PDOS peaks with severe changes and redistribution within them [43,44]. Therefore, it would be fair to state that pyridine oxime molecules, particularly 3POH molecules and the protonated form of 2POH, are chemically adsorbed on the iron surface, which is the main reason behind their corrosion inhibition performance [43].

### 2.2. Molecular Dynamics Simulation

Molecular dynamics simulation is a powerful way to analyze the adsorption of molecules on metal surfaces at a molecular level, which can further improve our theoretical insights and help to explain experimental performance [45]. These simulations, which are based on classical mechanics principles, can simulate the movement of atoms and molecules over time considering all contributing interactions between the molecules, the surface, and any other factors [46]. In corrosion studies, analyzing the trajectories of simulated inhibitor-metal systems in presence of a simulated solvent can provide useful insights into the molecular mechanisms that govern inhibitors’ adsorption, therefore providing additional knowledge to design new organic compounds with improved anti-corrosion properties.

**Figure 5:**
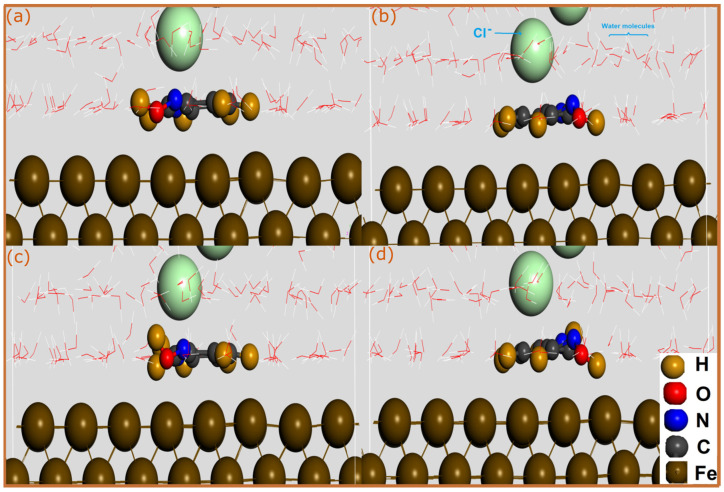
The most stable adsorption configurations of pyridine molecules adsorbed on Fe(110) surface in aqueous phase obtained by MD simulations; (**a**) 2POH, (**b**) 3POH, (**c**) 2POH^+^ and (**d**) 3POH^+^.

**Figure 6:**
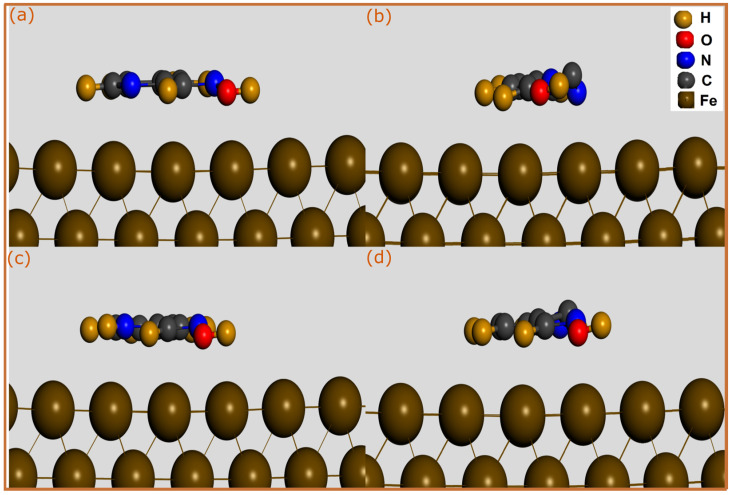
The most stable adsorption configurations of pyridine molecules adsorbed on Fe(110) surface in vacuum phase obtained by MD simulations; (**a**) 2POH, (**b**) 3POH, (**c**) 2POH^+^ and (**d**) 3POH^+^.

In this section, MD simulations were conducted to identify the most stable adsorption configurations of pyridine oximes on the Fe(110) surface in absence and presence of water molecules, chlorides, and hydronium ions at 303 K. The optimized adsorption configurations obtained by MD simulations for neutral and protonated pyridine molecules in aqueous states are illustrated in Figure 5. The vacuum state results are represented in Figure 6. It can be noticed from the adsorption configuration of both neutral and protonated forms that pyridine molecules exhibit a nearly parallel adsorption mode on the iron surface. When the molecule approaches the metal surface, it can experience electron exchange with the metal’s atoms by electron donation to its vacant orbitals or electron back donation to the anti-bonding orbitals of the molecule. The parallel adsorption mode can further maximize this adsorption process. In the presence of protonated molecules, the adsorption configuration seems to not be affected, maintaining the same parallel adsorption orientation on the Fe(110) surface. By inspecting the adsorption configuration of pyridine molecules in a vacuum (Figure 6), it is difficult to assess the difference in the interaction strength of molecules in aqueous and vacuum states given the similarity of the adsorption modes.

It is well-documented that classical molecular dynamics often overestimate the adsorption energy between a molecule and a metal surface due to some drawbacks related to used force fields such as the lack of electronic polarization effects and quantum-mechanical effects [44,47]. However, for the sake of comparison, it would be beneficial to calculate the adsorption energies of neutral and protonated pyridine oximes on a Fe(110) surface in vacuum and aqueous states. Table 2 lists the adsorption energies of neutral and protonated molecules of 2POH and 3POH compounds on a Fe(110) surface. Not surprisingly, it can be stated that the 3POH compound presents the highest adsorption strength in its neutral and protonated forms. The lower adsorption energies in the case of 2POH are attributed to its low affinity to iron surface. Additionally, it might also be because of its tendency to create non-bonding interactions with iron atoms such as van der Waals and electrostatic interactions. On the one hand, one can notice that the adsorption strength of the protonated 3POH molecule is slightly lower than its neutral counterparts. On the other hand, the interaction energies of pyridine molecules in a vacuum state are more negative than those in an aqueous state, as shown by the results in Table 2. In presence of a solution, water molecules and other particles can compete with the pyridine molecules for adsorption sites on the iron surface through the formation of hydrogen bonds with the molecule itself, therefore weakening the molecule–iron interactions. The results from MD simulation seem to support the conclusions arrived at from SCC-DFTB simulations.

### 2.3. Molecular Electrostatic Potential

**Figure 7:**
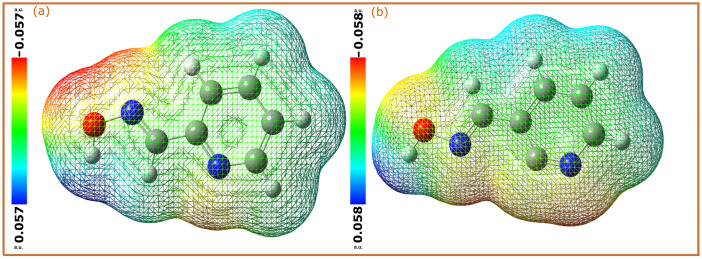
Molecular electrostatic potential of (**a**) 2POH and (**b**) 3POH molecules obtained by DFT/B3LYB method.

Molecular electrostatic potential (MEP) is a very useful visualization tool which is used to predict the reactivity of molecules [48]. In this case, the electron density is represented in the form of a color-coded map, making its polarization visible. A region with high electron density is mapped in red or orange, while a region with a low electron density is represented by blue areas [49]. In corrosion inhibition studies, these MEP maps can provide further insights into the electrophilic and nucleophilic behavior of molecules. The MEP maps of the 2POH and 3POH molecules are represented in Figure 7. By comparison, it can be seen that the pyridine moiety of 3POH is represented by a wide orange area around it, while it is only concentrated on the N atom of the 2POH molecule. In contrast, the -N-OH group of the 2POH molecule shows a very high electron density compared to that of the 3POH molecule. These high electron density areas of pyridine oximes are the potential binding sites with the iron surface, and therefore the main contributor to their corrosion inhibition performance [50].

## 3. Computational Details

### 3.1. SCC-DFTB Simulation

**Figure 8:**
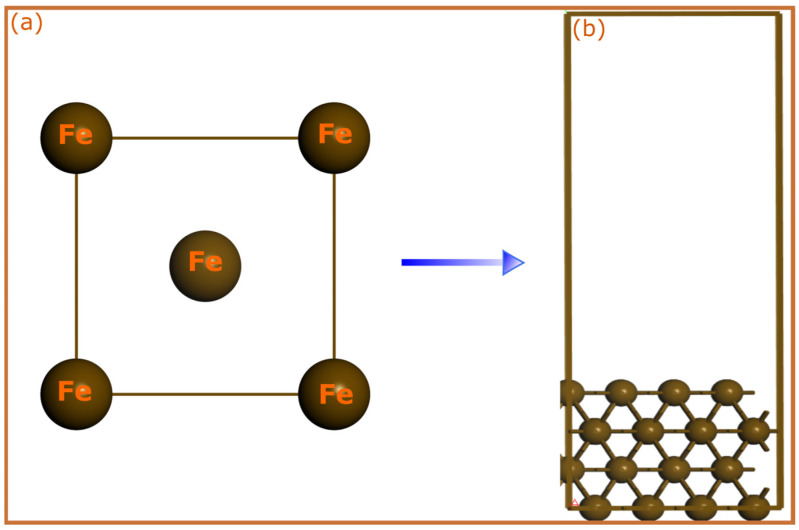
(**a**) iron crystal structure and (**b**) DFTB simulation box used for simulating pyridines adsorption on the iron surface.

The SCC-DFTB method is an approximation that assumes a second-order expansion of the Kohn–Sham Density Functional Theory. The SCC-DFTB approach has been benchmarked against DFT for organic-transition metal systems in several previous studies [51,52,53,54]. Its accuracy in predicting structural and electronic properties is similar to that of first-principles calculations, but it is faster, particularly for large systems. Herein, the inhibitor–iron interactions were fully optimized by the spin-polarized SCC-DFTB method, including dispersion interaction with Slater–Koster trans3d using the DFTB+ code [55]. The generalized gradient approximation (GGA) within its PBE formulation was used for the electron exchange and correlation [56]. The adsorption systems were fully optimized using a SCC tolerance of 10^−8^ au, thermal smearing (Methfessel-Paxton smearing distribution function), and Broyden mixing scheme. Meanwhile, the bulk lattice parameters’ optimization was carried out using a (8 × 8 × 8) *k*-point grid using ab initio DFT and SCC-DFTB for confirmation of suitability of the used model. The ab initio DFT calculations were performed using at GGA/RPBE level using CASTEP [57] code implemented in Materials Studio software 7.0 [58] to calculate lattice parameters. The iron crystal and simulation box used are shown in Figure 8. The optimization of bulk lattice parameters reproduced values of 2.884 Å (SCC-DFTB) and 2.857 Å (GGA/RPBE), which are close to the experimental value of 2.862 Å, confirming the accuracy of the selected parameters. The electronic energy of pyridine-Fe(110) surface was converged on (2 × 2 × 1) *k*-point grid. The adsorption models were generated by constructing Fe(110) iron surface consisting of a (3 × 3) supercell and a vacuum spacing of 20 Å along the z-direction separating periodic image in each direction. Then, neutral and protonated forms of molecules were placed on the top side of the slab and all atoms were allowed to relax except the two bottom-most atomic layers. A surface coverage of 1/9 ML was used for all simulations. SCC-DFTB optimization of standalone molecules was carried out by constructing a cubic box of 30 Å in size. The interaction energy was used as a main parameter to estimate the adsorption strength of molecules, which is calculated according to the following equation:

(1) $$E_{\mathrm{inter}}=E_{\mathrm{mol}/\mathrm{surf}}-\left(E_{\mathrm{mol}}+E_{\mathrm{surf}}\right)$$

where *E*_mol_, *E*_surf_, and *E*_mol/surf_ denote the total energies of isolated molecules, Fe(110) iron surface, and molecule/Fe(110) adsorption systems.

### 3.2. Molecular Dynamics Simulation

Molecular dynamics simulation can be used to aquire information about how strong the interaction of inhibitor molecules with the iron surface in presence of a simulated solution is [59]. To this end, an iron surface was constructed from a (110)-cleaved iron unit cell, extended to a (7 × 7) supercell and then placed in contact with a solvent layer consisting of one inhibitor molecule and 491 water molecules, 9 H_3_O^+^ and 9 Cl^−^. After the initial optimization of the constructed simulation boxes via Smart Minimizer protocol, the MD equilibrium process was carried out for 5000 ps in canonical ensemble (NVT) using COMPASSIII force field [60]. Furthermore, the summations techniques of Ewald and atom-based cut-off were used to treat non-bonded electrostatic and van der Waals interactions, respectively. The velocity Verlet integration scheme using a time step of 1 fs was used to solve the motion equation of Newton [61]. The temperature was controlled by a Nosé–Hoover thermostat at 303 K [62]. All MD simulations were carried out using a Forcite module implemented in Materials Studio software 7.0 [58]. The adsorption energies of molecules on Fe(110) surface were calculated from MD simulation by determining single-point energies of the complex adsorption system ($E_{\mathrm{total}}$), the metal surface without and with solution ($E_{\mathrm{surface}};\;E_{\mathrm{surface}+\mathrm{solution}}$), the inhibitor molecule without and with solution ($E_{\mathrm{molecule}};\;E_{\mathrm{molecule}+\mathrm{solution}}$), and the solution energy $\left(E_{\mathrm{solution}}\right)$ as follows:

(2) $$\mathrm{Vacuum}\;\mathrm{state}:\;E_{\mathrm{ads}}=E_{\mathrm{total}}-\left(E_{\mathrm{surface}}+E_{\mathrm{molecule}}\right)$$

(3) $$\begin{array}{l} \mathrm{Aqueous}\;\mathrm{state}:\;\;\\ E_{\mathrm{ads}}=E_{\mathrm{total}}-\left(E_{\mathrm{surface}+\mathrm{solution}}+E_{\mathrm{molecule}+\mathrm{solution}}\right)+E_{\mathrm{solution}} \end{array}$$

### 3.3. Quantum Chemical Calculations

Quantum chemical calculations were carried out to investigate the reactivity of individual inhibitor molecules. Full geometry optimizations of tested compounds were carried out using the density-functional theory (DFT), GGA functional and DNP basis set [56] using DMol3 code [63,64]. Computed quantum chemical parameters include the energy of the highest occupied molecular orbital (*E*_HOMO_), the energy of the lowest unoccupied molecular orbital (*E*_LUMO_), the energy band gap (Δ*E*g), and the fraction of transferred electrons (ΔN) among others. These quantum chemical reactivity indices are calculated via the following relationships as previously reported in the literature [65]:

(4) $$\mathrm{Energy}\;\mathrm{gap}:\;\Delta {Eg=E}_{\mathrm{LUMO}}-E_{\mathrm{HOMO}}$$

(5) $$\mathrm{Electronegativity}\;\mathrm{and}\;\mathrm{chemical}\;\mathrm{potential}:\;\chi =-\;µ=-\frac{E_{\mathrm{LUMO}}+E_{\mathrm{HOMO}}}{2}$$

(6) $$\mathrm{Hardness}:\;\eta =\frac{E_{\mathrm{LUMO}}-E_{\mathrm{HOMO}}}{2}$$

(7) $$\mathrm{Softness}:\;\sigma =\frac{1}{\eta }$$

(8) $$\mathrm{Electrophilicity}\;\mathrm{index}:\;\omega =\frac{\mu^{2}}{2\eta }$$

(9) $$\mathrm{Nucleophilicity}\;\mathrm{index}:\;\varepsilon =\frac{1}{\omega }$$

(10) $$\mathrm{Fraction}\;\mathrm{of}\;\mathrm{transferred}\;\mathrm{electron}:\;\Delta N=\frac{\chi_{\mathrm{metal}}-\chi_{\mathrm{mol}}}{2\left(\eta_{\mathrm{metal}}+\eta_{\mathrm{mol}}\right)}=\frac{\Phi -\chi_{\mathrm{mol}}}{2\eta_{\mathrm{mol}}}$$

where $\Phi_{Fe}$ = 4.82 eV, and the value of the total hardness $\eta_{Fe}$ of iron is taken to be zero.

Molecular electrostatic potential maps were calculated by DFT using B3LYB functional and 6-311G basis set using Gaussian 09W software [66] and visualized by GaussView 6.0 [67].

Fukui function indices were analyzed according to Hirshfeld population analysis using the following equations [68]:

(11) $$f_{k}^{+}=q_{k}\left(N+1\right)-q_{k}\left(N\right)$$

(12) $$f_{k}^{-}=q_{k}\left(N\right)-q_{k}\left(N-1\right)$$

(13) $$\Delta f\left(k\right)=f_{k}^{+}-f_{k}^{-}$$

where, *q_k_* (*N* + 1), *q_k_* (*N*), and *q_k_* (*N* − 1) are the electron densities on atom *k* corresponding to *N* + 1, *N* and *N* − 1 electrons systems, respectively.

Quantum chemical calculation and local reactivity results and discussion are represented in Supplementary Material.

## 4. Conclusions

In the present work, adsorption characteristics and bonding mechanisms of two pyridine oximes, namely 2-pyridylaldoxime (2POH) and 3-pyridylaldoxime (3POH), on a Fe(110) surface were investigated using quantum chemical calculations, molecular dynamics simulations and SCC-DFTB simulations. Atomistic simulations by SCC-DFTB revealed that the 3POH compound had a strong affinity to iron atoms through its neutral and protonated forms, whereas only the protonated form of the 2POH compound showed bonding ability with the iron surface. The order of the DFTB-calculated interaction energies successfully predicted the experimental corrosion inhibition performance of pyridine oxime, with 3POH having the strongest negative interaction energy of −2.534 eV, and 2POH (−0.007 eV) having the lowest. Quantum chemical calculations suggested that the bonding characteristics of the investigated molecules may be attributed to the difference in the chemical stability of the molecules according to energy gap values and the Hard and Soft Acids and Bases (HSAB) principle. Molecular dynamics simulation indicated that all neutral and protonated forms of pyridine oximes can exhibit a nearly flat adsorption mode on the metal surface in the absence and presence of a simulated solution. Computational insights from the present work could be useful knowledge-based information for the further design of pyridine-based corrosion inhibitors.

## Figures and Tables

**Table 1:** Interaction energies of neutral and protonated pyridine oximes adsorbed on a Fe(110) surface obtained by SCC-DFTB simulations (in eV).

Molecule	Interaction Energy
2POH	−0.007
2POH^+^	−1.897
3POH	−2.534
3POH^+^	−2.007

**Table 2:** Adsorption energies of neutral and protonated pyridine oximes adsorbed on Fe(110) surface obtained by MD simulation (in eV).

Molecule	Adsorption Energy	
	Vacuum State	Aqueous State
2POH	−10.258	−8.124
2POH^+^	−11.867	−11.047
3POH	−14.688	−13.745
3POH^+^	−12.744	−11.655

## Data Availability

The data presented in this study are available on request from the corresponding author.

## Supplementary Materials

The following supporting information can be downloaded at: https://www.mdpi.com/article/10.3390/molecules28083545/s1. Figure S1. DFT-optimized molecular structures (a,d), HOMO (b,e) and LUMO (c,f) maps of neutral pyridine oxime molecules obtained by DFT/GGA method. Figure S2. DFT-optimized molecular structures (a,d), HOMO (b,e) and LUMO (c,f) maps of protonated pyridine oxime molecules obtained by DFT/GGA method. Figure S3. Fukui function indices of neutral and protonated pyridine oximes obtained by DFT/GGA method; (a) 2POH, (b) 3POH, (c) 2POH+, and 3POH+. Table S1. Quantum chemical parameters of neutral and protonated pyridine oximes calculated using DFT/GGA method. References [69,70,71,72,73,74,75,76,77,78,79] are cited in Supplementary Materials.
