# Supplementary material for: Unraveling Bonding Mechanisms and Electronic Structure of Pyridine Oximes on Fe(110) Surface: Deeper Insights from DFT, Molecular Dynamics and SCC-DFT Tight Binding Simulations
Source: Molecules. 2023 Apr 18;28(8):3545. doi: 10.3390/molecules28083545 (PMC10141362; doi:10.3390/molecules28083545)
Supplement: Supplementary file 1 [file molecules-28-03545-s001.zip › molecules-2311446-supplementary-corr.docx]

Supplementary Materials

Unraveling Bonding Mechanisms and Electronic Structure of Pyridine Oximes on Fe(110) Surface: Deeper Insights from DFT, Molecular Dynamics and SCC-DFT Tight Binding
Simulations

Hassane Lgaz ^1^, Han-seung Lee ^2,*^, Savaş Kaya ^3^, Rachid Salghi^4,5^, Sobhy M. Ibrahim ^6^,
Maryam Chafiq ^7,*^, Lahcen Bazzi^5^ and Young Gun Ko ^7,*^

^1^ Innovative Durable Building and Infrastructure Research Center, Center for Creative Convergence Ed-ucation, Hanyang University ERICA, 55 Hanyangdaehak-ro, Sangrok-gu, Ansan-si, Gyeonggi-do 15588, South Korea

^2^ Department of Architectural Engineering, Hanyang University-ERICA, 55 Hanyangdaehak-ro, San-grok-gu, Ansan-si, Gyeonggi-do 15588, Republic of Korea

^3^ Sivas Cumhuriyet University, Health Services Vocational School, Department of Pharmacy, 58140, Sivas, Turkey

^4^  Laboratory of Applied Chemistry and Environment, ENSA, University Ibn Zohr, PO Box 1136, Agadir 80000, Morocco

^5^ Laboratoire de Génie Industriel, de l'Énergétique et de l’Environnement (LGI2E), SupMTI Rabat 10000 Morocco

^6^ Department of Biochemistry, College of Science, King Saud University, P.O. Box 2455, Riyadh 11451, Saudi Arabia

^7^ Materials Electrochemistry Group, School of Materials Science and Engineering, Yeungnam University, Gyeongsan 38541, South Korea;

***** Correspondence: ercleehs@hanyang.ac.kr (H-S. L.); maryam.chafiq@yu.ac.kr (M.C.); younggun@yu.ac.kr (Y.G.K.)

S1. Quantum chemical calculations

S1.1. Global reactivity descriptors

Quantum chemical calculations can complete our understanding on the adsorption characteristics of pyridine oximes under study. It can also reveal some structural and electronic features that would explain reasons behind the outstanding adsorption property of 3POH molecules compared to 2POH. Global reactivity descriptors such as the highest occupied molecular orbitals (HOMO), the lowest unoccupied molecular orbitals (LUMO), energy gap, ionization energy, electron affinity and fraction of transferred electrons can provide a way to assess the reactivity of individual molecules, making it easy to know their electronic features without external perturbation [18]. Before discussing numerical values of quantum chemical parameters listed in Table S1, it would be interesting to investigate the iso-surface maps of HOMO and LUMO of both neutral and protonated molecules depicted in Figure S1 and S2, respectively. HOMO and LUMO densities distribution reveal insights into the reactivity and chemical proprieties of molecules. The HOMO density distribution on a molecule or part of it indicates an electron-rich region where chemical reactions are likely to occur with other chemical species through electron donation [70]. On the other side, the LUMO density on a molecule or part of it indicates its tendency to accept electrons and undergo nucleophilic attack [71]. Looking at HOMO-LUMO maps of both neutral and protonated pyridine oxime molecules, it can be inferred that HOMO and LUMO densities are distributed over the entire molecular structures. It means that entire molecular structures could act as electron donating and/or electron accepting species when interacting with the metal surface. However, given the fact that it may be difficult for a molecule to enter into donor-acceptor interactions through its entire atomic sites, the HOMO-LUMO maps are confusing and cannot be used to distinguish between reactivity of investigated molecules. However, these maps can be represented in the form of energies, which would make them reasonably comparable.


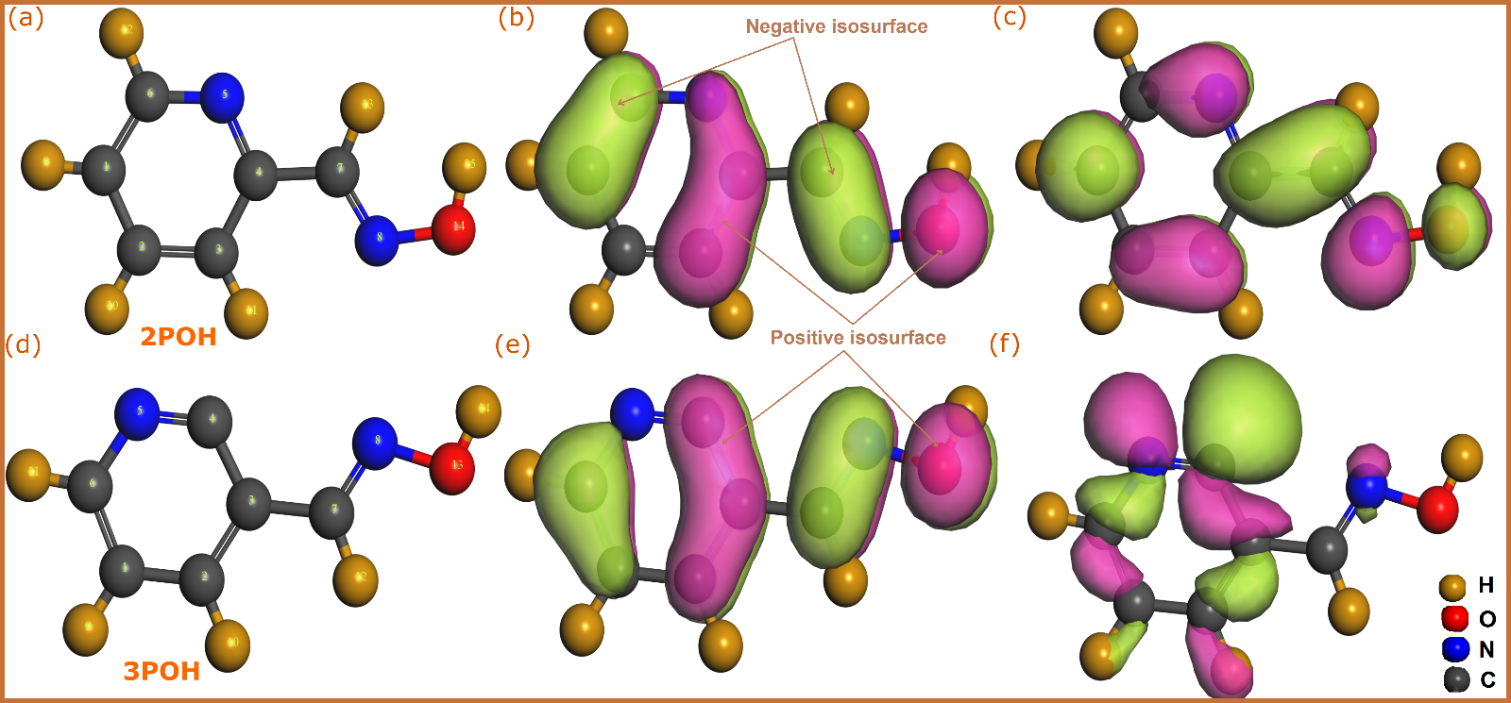


**Figure S1.** DFT-optimized molecular structures (a, d), HOMO (b, e) and LUMO (c, f) maps of neutral pyridine oxime molecules obtained by DFT/GGA method.


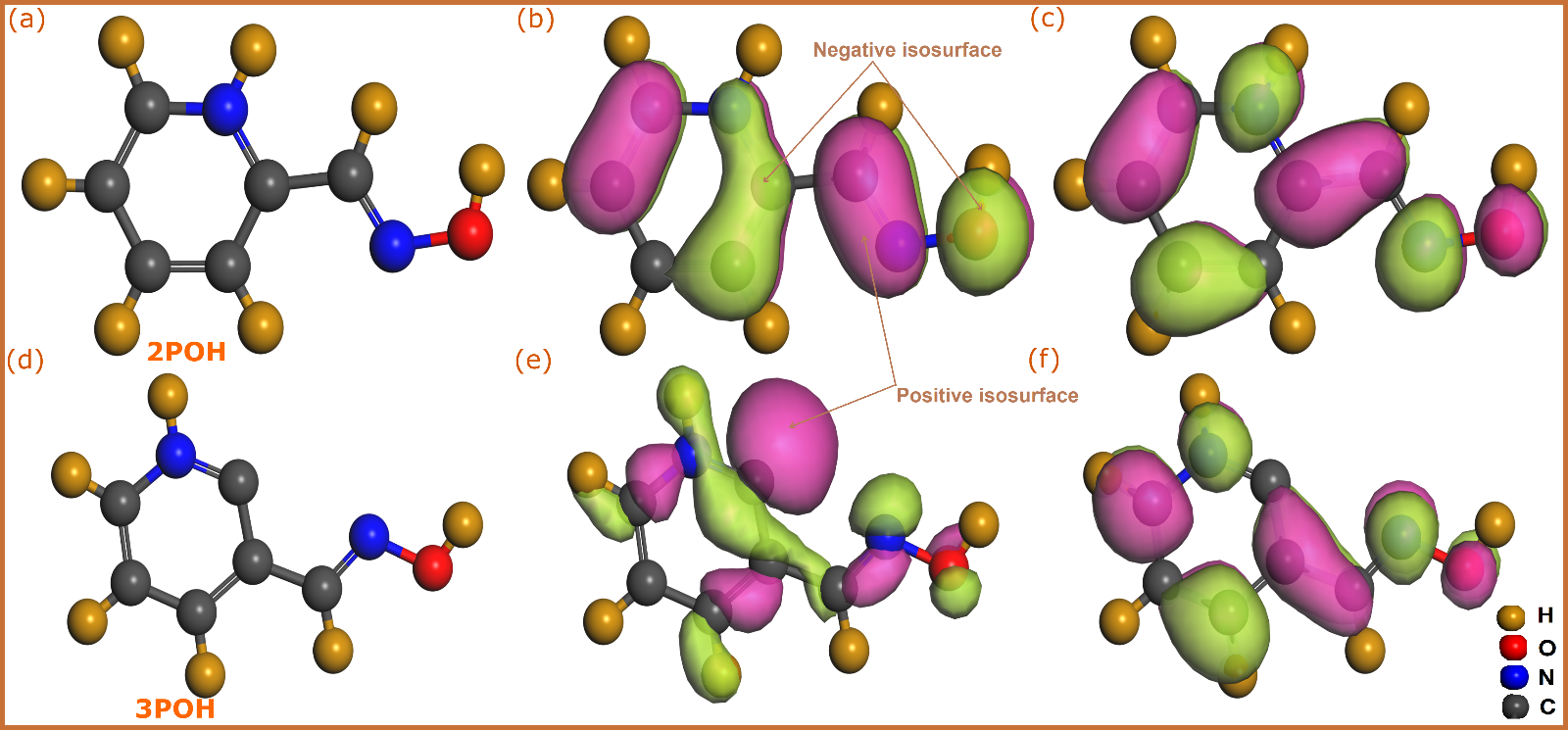


**Figure S2.** DFT-optimized molecular structures (a, d), HOMO (b, e) and LUMO (c, f) maps of protonated pyridine oxime molecules obtained by DFT/GGA method.

In HOMO and LUMO energies, noted *E*_HOMO_ and *E*_LUMO_, respectively, the same assumptions are made. A molecule with a higher HOMO energy is assumed to exhibit a higher electron donating ability, while a lower LUMO energy indicates its tendency to accept electrons [71]. Close inspection of quantum chemical parameters in Table S1 shows that 3POH molecule exhibits a higher tendency to donate and accept electrons given its higher HOMO energy and lower LUMO energy values compared to 2POH molecule [71]. In the protonated forms, the 3POH molecule keeps the highest electron donating ability while its electron accepting tendency is reduced compared with 2POH^+^ molecule. But both molecules show enhanced electron accepting and reduced electron donating tendencies after protonation.

**Table S1.** Quantum chemical parameters of neutral and protonated pyridine oximes calculated using DFT/GGA method.

| **Molecule** | ***E*_HOMO_** | ***E*_LUMO_** | **∆E** | ***Ƞ*** | ***ω*** | ***σ*** | **∆*N*** | ***ε*** |
| --- | --- | --- | --- | --- | --- | --- | --- | --- |
| 2PaOH | -6.091 | -2.66 | 3.431 | 1.715 | 5.580 | 0.582 | 0.129 | 0.179 |
| 3PaOH | -6.054 | -4.348 | 1.706 | 0.853 | 15.856 | 1.172 | -0.223 | 0.063 |
| 2PaOH+ | -6.874 | -3.753 | 3.121 | 1.560 | 9.046 | 0.640 | -0.158 | 0.110 |
| 3PaOH+ | -6.442 | -3.636 | 2.806 | 1.403 | 9.049 | 0.712 | -0.078 | 0.110 |

The chemical reactivity and stability of molecules can be discussed in view of their chemical hardness (*η*) and softness (*σ*), which are both correlated with the HOMO-LUMO energy gap (∆E) [72,73]. A molecule with a high chemical hardness (high energy gap) will require a lot of energy to undergo chemical changes or reaction with other species because of the higher energy barrier for electron transfer [74]. The chemical softness, on the other hand, is the inverse of the chemical hardness, and signifies the molecule’s susceptibility to undergo electron transfer with other chemical species, i.e., nucleophilic attacks [74]. Pearson has been reported that “*hard molecules have a large HOMO-LUMO energy gap and soft molecules have a small HOMO-LUMO gap*” [75]. According to results in Table S1, 3POH molecules have very small energy gap values, and therefore, it can be stated that neutral and protonated 3POH molecules are softer than 2POH molecules and expected to have very low stability, which would increase their reactivity with the iron surface.

Nucleophilicity (ε) and electrophilicity (ω) are two concepts that describe the ability of an electron-rich molecule to donate electrons and an electron-deficient molecule to accept electrons, respectively [74]. However, despite having similar concepts, these two parameters are not necessarily related to HOMO and LUMO energies given the contribution of other factors such as steric hinderance and the presence of functional groups. From results in Table S1, it can be observed that the neutral form of 3POH has a very high electrophilicity compared to 2POH molecule while the opposite is noticed in the case of nucleophilicity. In their protonated forms, both molecules exhibit similar nucleophilicity and electrophilicity indexes.

The fraction of electrons transferred (ΔN) is one of the well-known reactivity descriptors to describe the tendency of molecules to transfer their electrons to a metal surface. It has been reported that a positive ΔN value implies the ability of the molecule to transfer its electrons and vice versa when it is negative [76,77]. In the case of the present investigation, the calculated ΔN values indicate that the 2POH molecule has a more tendency to transfer its electron to the iron surface while the 3POH shows the opposite behavior. Therefore, it can be inferred that this parameter is unable to predict the same adsorption strength and bonding of tested compounds with the iron surface as revealed from experimental and simulation results.

According to quantum chemical parameters, it can be concluded that the reactivity of the investigated molecules can be successfully predicted based on "Hard-Soft-Acid-Base" (HSAB) principles. Based on this theory, it is found that the reactivity of both neutral and protonated molecules follows this trend: 3POH > 3POH^+^ > 2POH+ > 2POH, which is in line with the reactivity trend derived from SCC-DFTB simulations. The 3POH molecules are softer species and have low stability, and therefore tend to undergo electron transfer with electron-deficient iron atoms. It should be noted that there is no significant correlation between other quantum chemical parameters and experimental performance.

2.3.2. Local reactivity descriptors

Given the fact that the corrosion inhibition process is mostly electron-transfer controlled reactions, the investigation of local reactivity of molecules would lead to very beneficial conclusions about their interactions with iron atoms. Fukui functions, which has been introduced by Kenichi Fukui, are one of the well-recognized local reactivity descriptors [78]. This theory describes the behavior of electrons in a molecule when it undergoes a small perturbation [79]. Therefore, according to Fukui functions, it can be specified which atoms or a region in a molecule would act as nucleophilic site (electron donating tendency) or electrophilic site (electron accepting tendency). Fukui function indices of neutral and protonated pyridine oxime molecules are grouped in Figure S3. The site where the $f_{k}^{+}$ is maximum indicates a high electrophilic property (susceptible to nucleophilic attacks) while the highest $f_{k}^{-}$ means that the atomic site has a high nucleophilic character (susceptible to electrophilic attacks) [77]. Besides, Morell et al. have proposed a reactivity descriptor called dual descriptor to adequately describe the chemical reactivity of molecules given the fact that a molecule may act like a nucleophile and electrophile at the same time [79]. From results in Figure S3, one can notice that all atoms of 3POH molecule have almost equal electrophilic and nucleophilic properties. The C(4) atom, which is found to covalently bond with iron atoms, exhibits the highest reactivity followed by N(5) while all other atoms have similar reactivity characteristics. In the case of 2POH, the distribution of nucleophilic and electrophilic sites is significantly different. The reactivity of C(4) and N(5) is now considerably decreased while N(8) and O(14) atoms exhibit higher electrophilic and nucleophilic properties, respectively. When the 3POH gets protonated, the reactivity of C(4) and N(5) is drastically decreased, with slight improvement in reactivity of C(7), N(8) and O(13) atoms. In the case of the 2POH^+^ molecule, its electrophilic and nucleophilic characteristics are remarkably improved, but without changes in reactive sites distribution. Overall, results show that 3POH molecules have some strong reactive sites compared to 2POH molecules, which have almost equal distribution of reactive sites. Also, it can be noticed that 2POH molecules’ reactivity gets improved after protonation, which can explain their strong bonding ability with the iron surface. However, it is worth noting that it would be difficult to predict the binding sites of molecules for interaction with iron atoms from these near-equally distributed reactive sites.


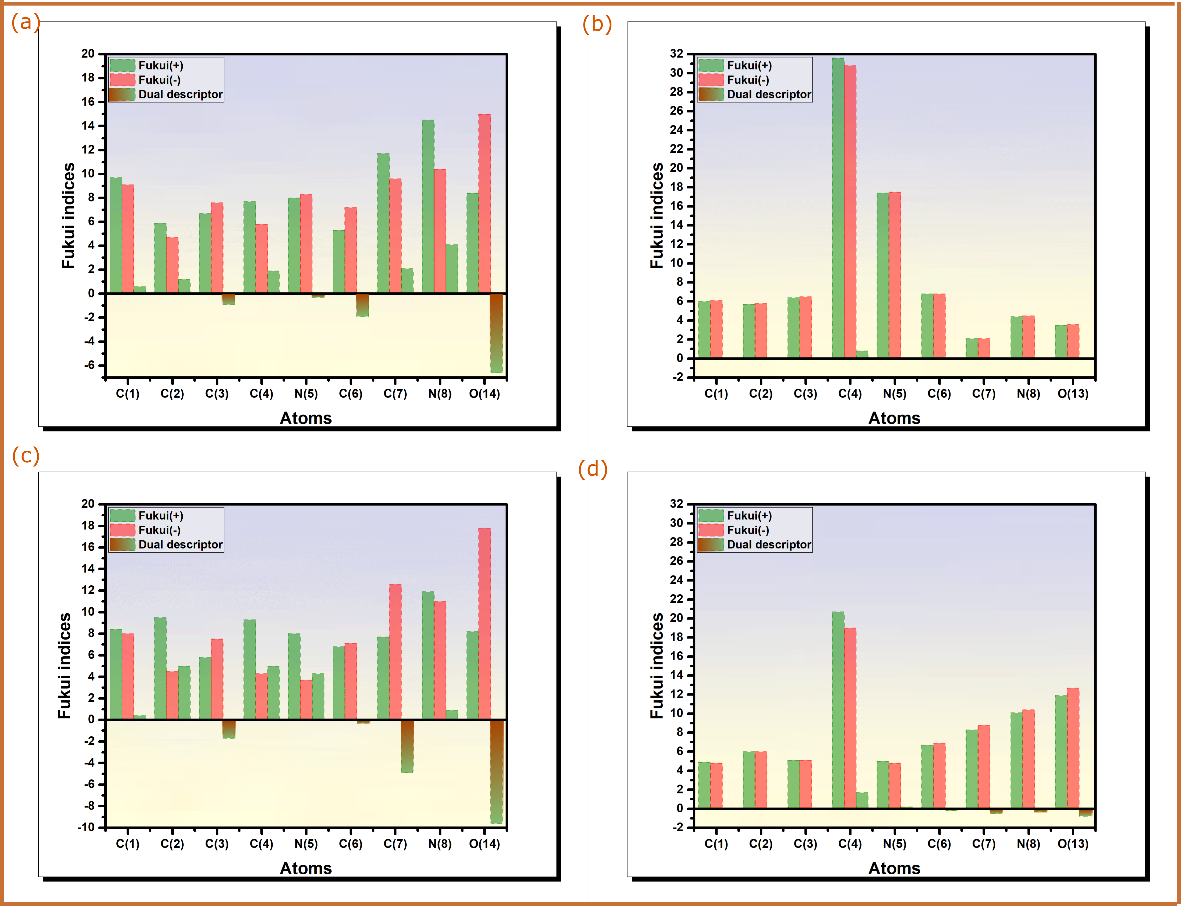


**Figure S3.** Fukui function indices of neutral and protonated pyridine oximes obtained by DFT/GGA method; (a) 2POH, (b) 3POH, (c) 2POH^+^, and 3POH^+^.

**References**

1. Obot, I.B.; Macdonald, D.D.; Gasem, Z.M. Density Functional Theory (DFT) as a Powerful Tool for Designing New Organic Corrosion Inhibitors. Part 1: An Overview. *Corrosion Science* **2015**, *99*, 1–30, doi:10.1016/j.corsci.2015.01.037.
2. Saha, S.K.; Murmu, M.; Murmu, N.C.; Banerjee, P. Evaluating Electronic Structure of Quinazolinone and Pyrimidinone Molecules for Its Corrosion Inhibition Effectiveness on Target Specific Mild Steel in the Acidic Medium: A Combined DFT and MD Simulation Study. *J. Mol. Liq.* **2016**, *224*, 629–638. https://doi.org/10.1016/j.molliq.2016.09.110.
3. Saha, S.K.; Murmu, M.; Murmu, N.C.; Banerjee, P. Benzothiazolylhydrazine Azomethine Derivatives for Efficient Corrosion Inhibition of Mild Steel in Acidic Environment: Integrated Experimental and Density Functional Theory Cum Molecular Dynamics Simulation Approach. *J. Mol. Liq.* **2022**, *364*, 120033. https://doi.org/10.1016/j.molliq.2022.120033.
4. Isin, D.O.; Karakus, N. Quantum Chemical Study on the Inhibition Efficiencies of Some Sym-Triazines as Inhibitors for Mild Steel in Acidic Medium. *J. Taiwan Inst. Chem. Eng.* **2015**, *50*, 306–313. https://doi.org/10.1016/j.jtice.2014.12.035.
5. Saha, S.K.; Murmu, M.; Murmu, N.C.; Obot, I.B.; Banerjee, P. Molecular Level Insights for the Corrosion Inhibition Effectiveness of Three Amine Derivatives on the Carbon Steel Surface in the Adverse Medium: A Combined Density Functional Theory and Molecular Dynamics Simulation Study. *Surf. Interfaces* **2018**, *10*, 65–73. https://doi.org/10.1016/j.surfin.2017.11.007.
6. Islam, N.; Kaya, S. *Conceptual Density Functional Theory and Its Application in the Chemical Domain*; CRC Press: Boca Raton, FL, USA, 2018; ISBN 978-1-351-36024-1.
7. Pearson, R.G. Hard and Soft Acids and Bases. *J. Am. Chem. Soc.* **1963**, *85*, 3533–3539.
8. Kokalj, A. On the HSAB Based Estimate of Charge Transfer between Adsorbates and Metal Surfaces. *Chem. Phys.* **2012**, *393*, 1–12. https://doi.org/10.1016/j.chemphys.2011.10.021.
9. Saha, S.K.; Dutta, A.; Ghosh, P.; Sukul, D.; Banerjee, P. Novel Schiff-Base Molecules as Efficient Corrosion Inhibitors for Mild Steel Surface in 1 M HCl Medium: Experimental and Theoretical Approach. *Phys. Chem. Chem. Phys.* **2016**, *18*, 17898–17911. https://doi.org/10.1039/C6CP01993E.
10. Makedonas, C.; Mitsopoulou, C.A. An Investigation of the Reactivity of [(Diimine)(Dithiolato)M] Complexes Using the Fukui Functions Concept. *Eur. J. Inorg. Chem.* **2006**, *2006*, 590–598. https://doi.org/10.1002/ejic.200500664.
11. Thanikaivelan, P.; Padmanabhan, J.; Subramanian, V.; Ramasami, T. Chemical Reactivity and Selectivity Using Fukui Functions: Basis Set and Population Scheme Dependence in the Framework of B3LYP Theory. *Theor. Chem. Acc.* **2002**, *107*, 326–335. https://doi.org/10.1007/s00214-002-0352-z.
12. Morell, C.; Grand, A.; Toro-Labbé, A. New Dual Descriptor for Chemical Reactivity. *J. Phys. Chem. A* **2005**, *109*, 205–212.
